# Catastrophic complications of PVL‐MRSA necrotizing pneumonia presenting as respiratory failure and rhabdomyolysis, case report and review of the literature

**DOI:** 10.1002/ccr3.6809

**Published:** 2023-05-16

**Authors:** Mouhammad J. Alawad, Sabeen Zara, Ahmad Elgohari, Abdulsalam Saif Ibrahim, Hamad Abdel Hadi

**Affiliations:** ^1^ Department of Medical Education, Internal Medicine Residency Program Hamad Medical Corporation Doha Qatar; ^2^ Division of Infectious Diseases, Department of Medicine Communicable Diseases Centre, Hamad Medical Corporation Doha Qatar; ^3^ Division of Medical Critical Care, Department of Medicine Hamad Medical Corporation Doha Qatar

**Keywords:** ARDS, extracorporeal membrane oxygenation (ECMO), methicillin resistant staphylococcus aureus (MRSA), Panton‐Valentine Leukocidin(PVL), rhabdomyolysis, staphylococcal aureus(SA)

## Abstract

Panton‐Valentine leucocidin toxin‐producing methicillin‐resistant staphylococcus aureus is an important uncommon cause of community‐acquired pneumonia; we describe a case of necrotizing pneumonia presenting as respiratory failure necessitating early initiation of extracorporeal membrane oxygenation, acute kidney injury and rhabdomyolysis, awareness, prompt recognition and appropriate management are crucial due to possible significant pathology.

## Graphical abstract

PVL‐toxin‐producing staphylococcal aureus necrotizing pneumonia should be suspected in previously healthy individuals presenting with severe community‐acquired pneumonia with multiorgan dysfunctions including respiratory failure and rhabdomyolysis.

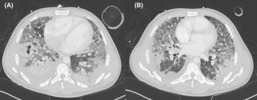

## 1 INTRODUCTION

Staphylococcal aureus (SA) is a major human bacterial pathogen associated with a wide disease spectrum ranging from skin and soft tissue infections to invasive disease encompassing infective endocarditis, osteoarticular and bloodstream infections as well as community and hospital‐acquired pneumonia.^1^ The historic pathogen is carved in the history of medical infectious diseases since the discovery of the first properties of the antimicrobial penicillin was established through the observation of its inhibition of SA colonies. Furthermore, the start of antimicrobial resistance was recognized a few years afterward when Methicillin Resistant Staph Aureus (MRSA) was first described.^2^

Over the last decades, community, as well as hospital‐acquired MRSA, emerged as a significant global threat to healthcare mainly because of rapid and ease of transmission, wide spectrum of disease presentation as well as significant morbidity and mortality.^3^ The distinction between community and hospital‐acquired MRSA is usually evident based on the affected population, microbiological characteristics as well as different clinical presentations.^4^ While community‐acquired MRSA (CA‐MRSA) tends to affect young and healthy individuals and is usually less resistant, hospital‐acquired disease (HA‐MRSA) tends to affect older patients, particularly those with chronic comorbidities as well as being more resistant and aggressive.^5^ The exception for community‐acquired SA infections is the rare but lethal toxin‐producing strains of Panton‐Valentine Leucocidin (PVL) SA that commonly present as recurrent skin and soft tissue infections and less frequently as invasive necrotizing pneumonia with ominous consequences. The PVL toxin‐positive strains manifested in <5% of isolates of both Methicillin Sensitive Staph Aureus (MSSA) as well as MRSA.^6^

The toxin was historically described more than a century ago but scientifically outlined by Panton and Valentine in their seminal paper in 1932.^7^ The cytopathic toxin is composed of two subunits LukS‐PV and LukF‐PV which are secreted separately by the pathogen but assembled as a pore‐forming heptamer at cellular membranes leading to instant lethal damage with secondary inflammatory responses.^8^ The PVL toxin has a strong pathogenic predilection for leukocytes hence the historic nomenclature. Since neutrophils of leukocytes are the major component of the innate immunity system triggered to contain invading pathogens, their early destruction by the toxins leads to halting disease eradication and progression. More importantly, the presence of the PVL toxin in addition to other SA toxins enhances the lethal and destructive nature of the organism towards epidermal and underlying tissues.^9^

Additionally, Rhabdomyolysis (RM) is a clinical syndrome that is associated with myonecrosis leading to the release of muscular cell contents including myoglobin and the enzyme creatinine phosphokinase (CPK) frequently leading to substantial disease manifestations.^10^ Renal tubular lumen blockage might lead to secondary acute kidney injury (AKI) usually managed conservatively or in severe cases through transient renal replacement therapy to accommodate for the sudden loss of renal function.^10^ Among non‐traumatic causes of RM, drugs, and infections are the major precipitating causes. Various viral, parasitic and bacterial infections have been implicated encompassing gram‐positive and negative bacteria including SA.^11^, ^12^

We present a rare case of young healthy adults patients with PVL‐producing MRSA infection presented as acute necrotizing pneumonia that progressed within days into an Acute Respiratory Distress Syndrome (ARDS) that necessitated Extracorporeal Membrane Oxygenation (ECMO) accompanied by rhabdomyolysis that progressed into AKI managed with renal replacement therapy (RRT). We will outline disease presentation, clues to early recognition, and the role of specific antitoxin therapy, including immunoglobulins.

Since the disease is associated with significant morbidity and mortality, we endeavor to raise awareness among front‐line physicians who might encounter the same disease presentation as well as highlight public health issues related to the disease.

## 2 CASE PRESENTATION

A 24‐year healthy male who is previously fit and well presented to the emergency department with 4 days history of progressive chest pain, productive cough of yellow sputum with streaks of hemoptysis, and shortness of breath accompanied by fatigue, myalgias, and arthralgia. The patient had no significant past medical or recent travel history and no apparent risk factors for drug misuse or other social habits.

Upon initial assessment the patient was clearly in critical distress, evidenced by tachycardic with a heart rate of 120 BPM, hypotension with a blood pressure of 90/50, and respiratory compromise with a respiratory rate of 35 BPM and oxygen saturation of 90% in room air only improved to 95% following 6 liters of oxygen using a facial mask. The initial temperature was not elevated at 36.8°C while the cardiovascular examination was unremarkable but pulmonary evaluation revealed significant pathology with extreme tachypnoea and hypoxia, decrease air entry to lung bases, and diffuse bilateral coarse crackles. The rest of the general examination was unremarkable although the patient had subjective muscular pains, objectively, the neurological and musculoskeletal evaluation did not elicit any deficits. Because of the gravity of the patient's condition, he was promptly resuscitated with fluids management, oxygen supplantation as well as broad‐spectrum antimicrobials namely Piperacillin‐tazobactam and Azithromycin following obtaining needed evaluation tests.

**FIGURE 1:**
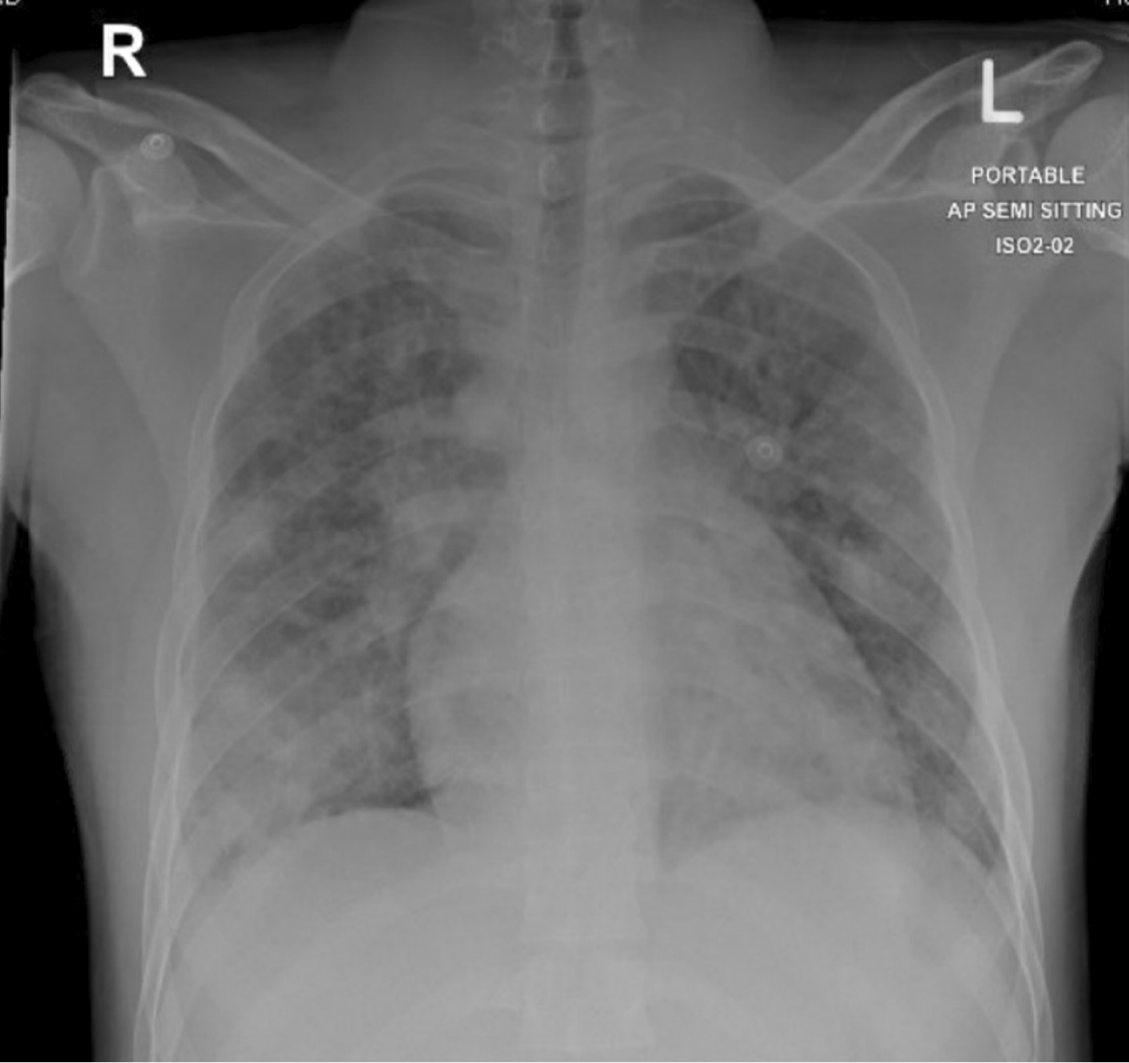
Anterior‐posterior chest XR, showing bilateral diffuse airspace opacities

**FIGURE 2:**
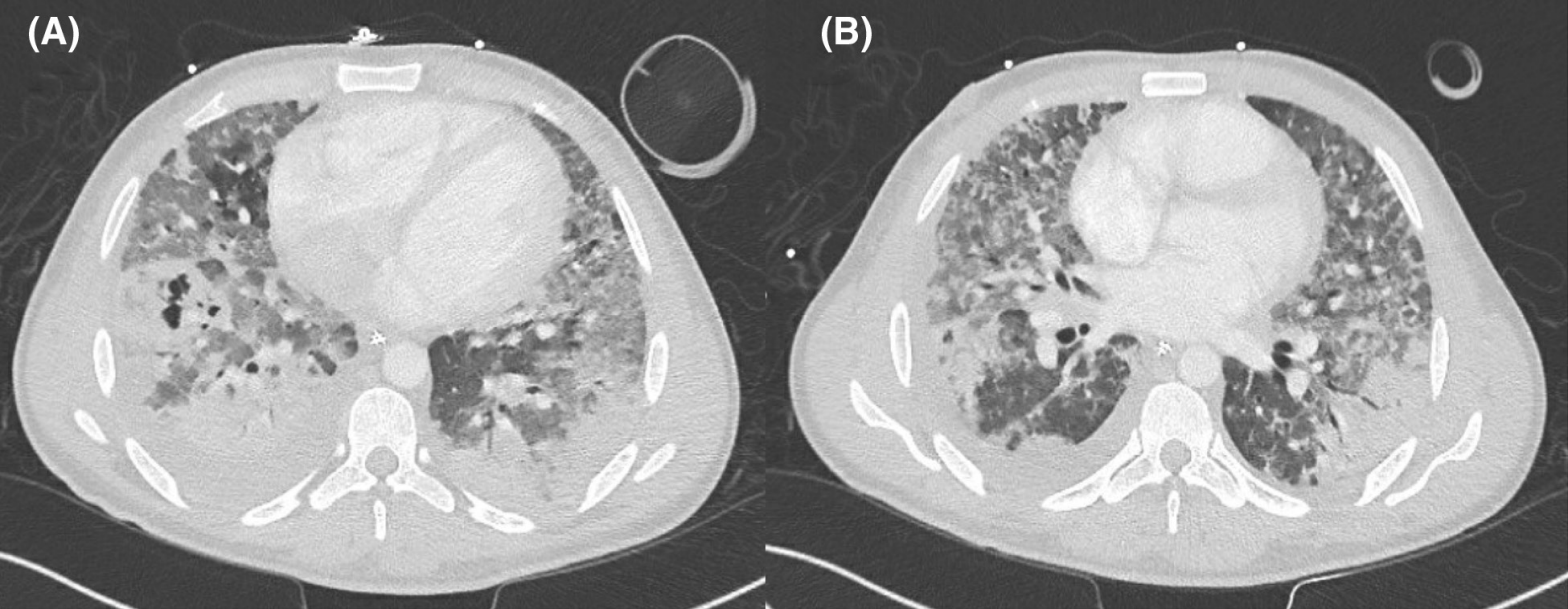
A,B: CT chest, showing diffuse bilateral opacification of both lungs fields with multiple areas of patchy consolidations with air bronchogram and areas of necrosis

Laboratory results showed hemoglobin of 14.2 gm/dL, significant leukopenia with a white blood count of 2.9 x 10^3/uL (absolute neutrophil count of 78%), thrombocytopenia with platelets count of 102 μl declined to a nadir of 20 μl, Acute Kidney Injury (AKI) with the creatinine of 169 umol/L and blood urea 12.9 mmol/L, while electrolytes were normal with Na 135 mmol/L and K 3.8 mmol/L. Inflammatory markers were markedly elevated validated by C‐reactive protein of 307.5 mg/L, and of procalcitonin of 8.83 ng/mL. Evaluation for the AKI revealed markedly elevated Creatinine Phosphokinase (CPK) at 1282 U/L, with raised myoglobin levels at 1019 ng/mL and a positive urine test. The viral panel was negative, while the initial chest X‐ray was grossly abnormal (Figure 1) showing diffuse bilateral rounded infiltrative opacities suggestive of bilateral pneumonia. The initial evaluation was of severe community‐acquired pneumonia subsequently became complicated when blood cultures grew Methicillin Resistant Staph Aureus (MRSA) 10 h into admission hence antimicrobial cover was broadened with the addition of vancomycin. The diagnosis of community‐acquired MRSA pneumonia was augmented with positive sputum cultures as well as MRSA screening tests, both were positive. Trying to detect any potential underlying causes for the critical presentation, both transthoracic and trans‐esophageal echocardiograms failed to identify potential infective endocarditis while the HIV test was negative as well as urinary legionella antigens. To expand evaluation, the CT chest demonstrated severe bilateral bronchopneumonia with significant destructive consolidation, while abdominal CT revealed a small right gluteal collection probably from the previous intramuscular injection which was sterile upon aspiration (Figure 2).

**FIGURE 3:**
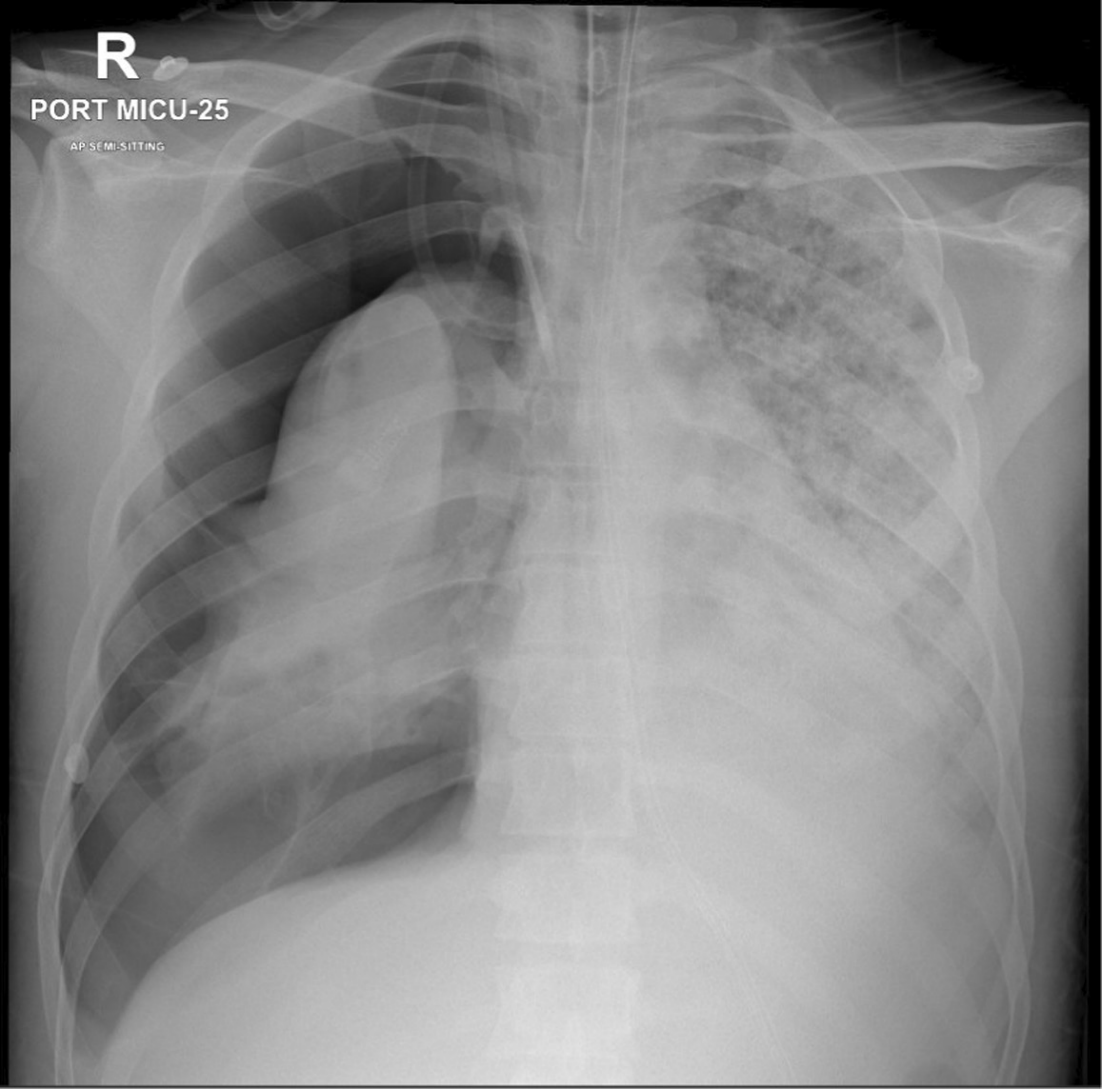
Progression of the necrotizing pneumonia showing the development of right sided pneumothorax

Because of the critical presentation, the patient was admitted to critical care for supportive care but deteriorated the following day with increased oxygen requirements confirmed with blood gases hypoxemia and acidemia necessitating mechanical ventilation but continued to deteriorate with the rapid development of bilateral pneumothoraxes on the third day (Figure 3). This complicated the existing respiratory failure to establish acute Adult Respiratory Distress Syndrome (ARDS), hence Extracorporeal Membrane Oxygenation (ECMO) was initiated accordingly. Additionally, the associated rhabdomyolysis and hypotension resulting in AKI were managed by intermittent renal replacement therapy (RRT) for 1 week, then his kidney parameters started to improve.

Suspecting PVL‐producing MRSA, high‐dose clindamycin was added at 900 mg QDS together with two doses of intravenous immunoglobulins as salvage therapy, confirmed by a positive PVL‐PCR on the third day of admission. Because of the profound thrombocytopenia, Linezolid was initially withheld and then added cautiously upon platelet count recovery. Despite all these measures it took almost 10 days to control the recurrent bacteremia, while RRT was administered for 7 days and the patient was ECMO‐dependent for the following 90 days. The patient remained alive but was left with significant morbidity which required extensive rehabilitation therapy and prolonged hospital stay.

## 3 DISCUSSION

Depending on susceptibility to penicillin‐based antimicrobial class, Staphylococcal aureus (SA) is sub‐classified into Methicillin sensitive (MSSA) and Methicillin Resistant (MRSA) both with divergent microbiological characteristics as well as clinical presentations and outcomes.^1^ Furthermore, community and healthcare‐associated SA infections have distinct characteristics evident for both pathogen qualities and host risk factors. Since the 1990 s community‐acquired SA infections particularly CA‐MRSA posed a significant public health threat rising to the public domains leading to a wide disease spectrum as well as frequent outbreaks.^4^ One of the rare but key factors for the disease morbidity is the production of PVL‐toxins by both community MSSA and MRSA strains. The postulation that the virulent toxin was initially embedded into MSSA SA through bacteriophages explains its presence in both community MSSA as well as MRSA leading to increased pathogenicity.^6^

The disease is commonly associated with recurrent skin and soft tissue infections (SSTIs) since it has a direct cytopathic effect on host epidermal cells as well as leukocytes which are attracted to contain the invading pathogens.^9^ Besides SSTIs, PVL‐positive SA is also capable of causing rare but complicated bone and bloodstream infections, fulminant sepsis as well as necrotizing pneumonia.^13^

There are global variations in the epidemiology of the disease, while PVL‐positive CA‐MSSA is more common in some countries like the UK, PVL CA‐MRSA is more predominant in many countries such as the USA, Asia including the Middle East usually linked to specific epidemic clones.^14^ The disease is usually suspected when patients present with multiple, recurrent and fleeting skin and soft tissue infections that are difficult to manage with standard interventions such as surgical drainage and appropriate antimicrobials coverage.^15^ When the local epidemiology does not support such observations, exploring travel history is mandatory since the disease is commonly imported.^14^ In rare situations, an invasive disease can supervene leading to septicemia or necrotizing pneumonia (NP) with ominous consequences. As outlined in Table 1, recognition clues for NP include high fever upon presentation, chest pains, cough associated with hemoptysis, and rapid and toxic deterioration. Laboratory test frequently demonstrates anemia, leukopenia, and thrombocytopenia as well as the radiological finding of diffuse lung involvement. The disease progression of NP leads to pulmonary necrosis, gangrene, and cavitations with frequent pneumothoraxes. Despite early recognition, of PVL‐associated NP, rapid deterioration might occur secondary to pulmonary insults frequently leading to respiratory failure and ARDS necessitating critical interventions including ECMO management.^16^ In our presented case all these clinical entities were present during the evaluation and the course of management.

**TABLE 1:** To aid in early recognition of early PVL‐associated necrotizing pneumonia: key parameters of clinical, laboratory and radiological evaluation clues are suggested, these values were adapted upon reviewing published literature, the parameters mainly correlate with systemic inflammatory response and severe sepsis.

Symptoms	Signs	Diagnostic tests	Radiological imaging
Fever	High grade fever >39°C	Marked leukopenia Anemia and thrombocytopenia	Chest Xray: Multi‐lobar infiltrates with or without cavitatory lesions, usually accompanied by pleural effusions
Cough with hemoptysis Extreme shortness of breath	Tachypnea RR > 40 per minute	Significantly high C‐Reactive protein (CRP) in the range of 200–350 mg/L	CT Scan: diffuse bilateral multi‐lobar consolidation with or without cavitations and often pneumothoraces
Toxic presentation	Tachycardia, Pulse >140 BPM Early hypotension SBP < 100 mmHg Features suggestive of toxic shock	Sputum microscopy: sheets of gram‐positive cocci	Early progression into acute respiratory distress syndrome (ARDS)
Extreme myalgia	Muscular tenderness	High creatine phosphokinase levels (CPK)	
Influenza like illness (ILI)	Absence of upper respiratory pathology	Early positive blood cultures showing gram positive cocci in clusters confirmed as Staphylococcal aureus	
Gastrointestinal symptoms: nausea, vomiting and diarrhea	Features suggestive of multiorgan involvement	Molecular identification of PVL gene by PCR	

Adapted from MS Morgan.^8^

It is intriguing to examine associated rhabdomyolysis (RM) in the context of the patient assessment. Upon presentation, the patient complained of intense myalgia which wasn't supported objectively by musculoskeletal deficit, nevertheless, his renal function was impaired coupled with very high levels of creatinine phosphokinase (CPK) and myoglobin levels. The combination of muscular symptoms accompanied by high CPK above and myoglobinuria usually establish RM.^10^ sepsis is an important risk factor for non‐traumatic rhabdomyolysis with the lung being the most commonly encountered source of infection.^12^ Various infectious pathogens like viral infection with influenza A, B, coxsackievirus, adenoviruses, and others were described in the context of RM.^11^ Adenoviruses have been implicated in a severe form of pneumonia with acute respiratory distress syndrome (ARDS) and respiratory failure (RF) with a high mortality rate, especially in immunocompromised populations like neonates or bone marrow transplant patients.^17^, ^18^ The possible link between adenoviral infection – especially if presenting as pneumonia or disseminated infection – and the development of RM could be established in further studies. For bacterial pathogens, Gram‐negative bacteria have been more commonly associated when compared to gram‐positive bacteria but only a few cases of SA have been reported. Similarly, pneumonia secondary to bacterial infection is linked to pneumococcal disease, mycoplasma, or legionella but rarely to SA.^12^ SA‐associated myositis or necrosis might be implicated since there are no available reports in the literature of direct PVL‐associated RM although the condition is frequently described in the context of severe sepsis or toxic shock secondary to PVL SA disease.^8^

When suspecting PVL‐NP, it is important to carry out parallel confirmatory and appropriate management as early as possible. Molecular tests such as PCR are the hallmark to confirm the disease for the detection of the toxin while anti‐toxin agents are the most recommended therapy.^8^ Antimicrobials that inhibit protein synthesis thus toxin production such as high‐dose clindamycin and Linezolid, have been advocated to combat toxin‐related pathology but caution must be extorted related to the context of evaluation.^19^ Being a bacteriostatic drug frequently leading to bone marrow suppression, linezolid is not usually advocated for MRSA bacteremia nor for patients with profound bone marrow suppression including severe anemia and thrombocytopenia.^20^ Conversely, the large molecular size of glycopeptides such as vancomycin precludes penetration into lung tissues leading to lower concentrations while linezolid has a higher tissue bioavailability. This contrasting variability has been examined widely when linezolid has been shown to manifest better microbiological eradication but both agents were comparable in outcomes.^21^

Furthermore, early adjuvant immunoglobulins in single or dual doses have been recommended to treat severe and critical diseases. The background stems from the presence of PVL‐positive immunoglobulins in healthy individuals that can chelate and neutralize circulating PVL toxins.^22^ Although there is no clear evidence based on clinical trials of its supportive role, it has been advocated based on animal studies as well as case series.^23^

Of note, although the mainstay of management focuses on the management of secondary complications, it is imperative to search for primary source identifications, especially in severe and refractory cases. Echocardiograms are mandatory for SA bacteremia to exclude associated infective endocarditis while careful assessment for complicated SSTIs sources is needed for source control. In our case, the source of infection was a dilemma, till the history of the intramuscular infection was revealed and confirmed upon radiological imaging. From previous studies, PVL‐associated pneumonia has been reported following SSTIs.^24^

## 4 CONCLUSION

Although community‐acquired pneumonia secondary to staph aureus infections is becoming increasingly common; PVL‐producing SA is associated with significant morbidity and mortality with ominous complications that include sepsis, acute kidney injury, and respiratory failure. The disease should be suspected in young and healthy individuals presenting with a toxic presentation with multiorgan involvement. Combination therapy with antitoxin agents such as clindamycin and linezolid is recommended in addition to the possible need for immunoglobulin therapy. Despite the best interventions, catastrophic complications might not be prevented.

## AUTHOR CONTRIBUTIONS

**Mouhammad J Alawad:** Conceptualization; data curation; validation; visualization; writing – original draft; writing – review and editing. **Sabeen Zara:** Conceptualization; data curation; formal analysis; resources; writing – review and editing. **Ahmad J Elgohari:** Conceptualization; data curation; formal analysis; writing – review and editing. **Abdul Salam S Ibrahim:** Conceptualization; supervision; validation; visualization; writing – review and editing. **Hamad Abdel Hadi:** Conceptualization; supervision; validation; visualization; writing – review and editing.

## FUNDING INFORMATION

No funding was received, Open access fees were provided by Qatar National Library.

## CONFLICT OF INTEREST

The authors declare no conflict of interest.

## ETHICAL APPROVAL

This case was approved by the Medical Research Center of Hamad Medical Corporation MRC‐04 – 22 – 448, and the patient consented to the publication of his case.

## CONSENT

Written informed consent was obtained from the patient to publish this report in accordance with the journal's patient consent policy.

## Data Availability

Data and materials are available on request.
